# Editorial: FEMS EC Thematic Issue “Aquatic Microbial Ecology”

**DOI:** 10.1093/femsec/fiaf005

**Published:** 2025-01-29

**Authors:** Martin W Hahn, Veljo Kisand

**Affiliations:** Research Department for Limnology, University of Innsbruck, Mondseestrasse 9, A-5310 Mondsee, Austria; Centre for Limnology, Institute of Agricultural and Environmental Sciences, Estonian University of Life Sciences, Rannu, Tartu County, Estonia; Institute of Technology, University of Tartu, Tartu, Estonia

## Abstract

High relative abundance of yeast (Vishniacozyma, Papiliotrema, and Filobasidium) and bacterial (Massilia) genera found in spinach seeds correlates with suppression of Globisporangium ultimum damping-off infection.

About 71% of the Earth’s surface is covered by liquid water with >99% of this area represented by oceans. Surface freshwater systems, i.e. rivers, lakes, and ponds, represent only about 0.5% of the Earth’s surface area. A much larger amount of liquid freshwater than collectively contained in those surface systems is present as ground water beneath the Earth’s terrestrial areas. These marine and freshwater systems are for a multitude of reasons of high or even crucial importance for the human population on the Earth. This includes reasons such as climate change, drinking water production, irrigation of crops, fisheries, and aquaculture. All these interests are linked to ecosystem services provided by those marine and freshwater systems. It is well known that microbes play central roles in the metabolism of all those systems; thus, they are crucially involved in most of the ecosystem services provided by aquatic systems. While the crucial involvement of microbes is a well-accepted fact, many details about microbial diversity and their roles in the metabolism in aquatic systems are not well understood. In addition, it is not well known how the increasing anthropogenic impact is influencing microbial communities and how this might change ecosystem services.

The Symposium for Aquatic Microbial Ecology (SAME17), which took place in Tartu, Estonia, on 20–25 August 2023, marked a significant milestone in the field, bringing together 135 participants. Among the attendees were 43 Early Career Researchers (ECRs) who played a vital role in the symposium’s success. The SAME17 was a testament to the global appeal of aquatic microbial ecology. Researchers, scholars, and experts from over 20 countries gathered to exchange knowledge and foster international collaboration. The theme of the conference was “From Isolation to Collaboration”. This diverse representation underlined the symposium’s significance on the global stage.

At the plenary session, Prof. Pei-Yuan Qian highlighted interactions within microbial communities and Prof. Meinhard Simon focused on the intricate processes by which marine bacteria cycle essential vitamins, highlighting the roles of both vitamin-producing (prototrophic) and vitamin-requiring (auxotrophic) bacteria in marine ecosystems.

Several key topics were discussed, reflecting the current diverse and dynamic nature of aquatic microbial ecology. The main themes were as follows: Carbon Cycling: Jan-Hendrik Hehemann focused on the role of microbes in carbon cycling; Nitrogen and Phosphorus Cycles: Lasse Riemann discussed the critical roles these elements play in aquatic ecosystems; Aquatic Microbial Eukaryotes: Ramiro Logares explored the diversity and function of these organisms; Microbes in a Changing World: Silke Langenheder addressed how environmental changes impact microbial ecology; Ecology of Aquatic Fungi: Keynote by Silke Van den Wyngaert focused on the role of fungi in aquatic environments; Deep Aquatic Biosphere: Anaïs Cario discussed microbial life below 200 m in the water column and subsurface environments; Big Data and Data Re-use: Lucas Paoli highlighted the importance of big data and the reuse of data in advancing aquatic microbial ecology; Microbes in a Changing World—Historic Perspective: Raffaele Siano provided a historical perspective on how environmental changes have impacted microbial ecology; Waterbody–Land–Atmosphere Interaction: Thorsten Brinkhoff explored the interactions between water bodies, land, and the atmosphere in microbial ecology. In addition, Early Career Researchers Session provided a platform for emerging scientists to present their research and network with established experts.

This thematic issue presents 13 contributions spanning wide ranges regarding microbes, habitat types, and ecological processes investigated. Both marine and freshwater systems are rather equally covered by the presented studies. A couple of the included studies focus on processes at the base of aquatic food webs, i.e. on factors regulating primary production and remineralization.

Waggoner et al. (2024) studied the potential of marine *Synechococcus* spp. to replace the common phosphate source orthophosphate by organic phosphorous compounds. Piwosz et al. (2024) investigated how the availability of dissolved organic substrates influenced the growth and competitiveness of aerobic anoxygenic phototrophic bacteria, which are potentially able to grow as photoheterotrophes. While the former study was conducted by using axenic cultures of the phototrophs, the latter investigation was performed by whole community manipulation experiments.

The study by Vanharanta et al. (2024) is focused on processes coupling primary production and remineralization taking place in a marine environment during the decline of a spring phytoplankton bloom. Two studies focus on potentially harmful cyanobacterial blooms and the interaction of the bloom-forming phototrophs with the associated heterotrophic bacterial communities. The papers by Jin et al. (2024) and Underwood et al. (2024) both aimed at revealing microbial interactions antagonistic to or supporting the bloom-forming cyanobacteria but focused on different habitats. The investigated lake systems have a lot in common such as eutrophic status, similar lake size, location at similar altitudes but with different climatic conditions, and in the cyanobacterial species forming the blooms. While one study performed a rather deep analysis of 16S rRNA gene amplicon data obtained by cultivation-independent methods, the other study combined analysis of amplicon data with experimental investigations on the influence of particular microbes on the growth of the bloom-forming cyanobacterium. The study by Karlicki et al. (2024) investigated the relationship between free-living bacteria and small protists in a temperate lake with a spatio-temporal resolution. As in the former studies, various interactions between the two groups of organisms may take place, but, in addition, predator–prey interactions have to be expected. One more study investigated the interaction of phototrophs with other organisms. In this case, Biggs et al. (2024) investigated the influence of temperature on the fate of Antarctic phytoplankton along two possible routes, microplankton predation and viral lysis, which potentially differ in the availability of phytoplankton carbon for other levels of the food web. Two other studies investigated microbes thriving in cold environment. Han et al. (2024) investigated the interaction between bacteria and planktic algae in Arctic fjords under the influence of melting glaciers. In the second case, Guo et al. (2024) investigated spatial differences in bacterial community structures in a lake caused by the impact of glacial and non-glacial streams discharging into the lake. Verma et al. (2024) investigated how growth conditions (simulated winter and summer conditions) influenced resource allocation, gene expression, and morphological features of marine bacterioplankton. Excitingly, the growth conditions were even reflected at the morphological level.

The paper by Diver et al. (2024) deals with rather rarely studied components of marine nanoplankton. They characterized nitrogen-uptake capabilities and growth responses of marine yeasts and revealed for one yeast genus a global open ocean distribution. Other rather rarely studied microbes are bacteria dwelling in aquifer systems (ground water systems), especially when obtained as pure cultures. The study by Garry et al. (2024) characterizes the physiology of a slowly growing autotrophic bacterium obtained from an aquifer system. Such investigations are challenging by the delicate nature of such organisms especially when only growing as liquid cultures.

The study by Duval et al. (2024) focused on rather small, mainly artificial freshwater habitats and addressed the reciprocal relationship between physico-chemical water quality (including pollution), microbial community composition, and absence/presence of mosquito larvae (*Aedes albopictus*).

In summary, the publications collected in the Thematic Issue “Aquatic Microbial Ecology” reflect the enormous diversity of freshwater habitats on the Earth ranging from tiny man-made habitats such as containers collecting rainwater to lakes or aquifer systems and further to huge open ocean systems. All these systems are of relevance for humans, for instance, due to mosquitoes emerging from such systems and potentially transmitting pathogens or due to climatic impact. On the other hand, the included papers well demonstrate the complexity of microbial communities and the multitude of types of interactions between different microbes or groups of microbes within each of these communities.
